# Heparanase mediates renal dysfunction during early sepsis in mice

**DOI:** 10.1002/phy2.153

**Published:** 2013-11-11

**Authors:** Melissa I Lygizos, Yimu Yang, Christopher J Altmann, Kayo Okamura, Ana Andres Hernando, Mario J Perez, Lynelle P Smith, Daniel E Koyanagi, Aneta Gandjeva, Rhea Bhargava, Rubin M Tuder, Sarah Faubel, Eric P Schmidt

**Affiliations:** 1 Program in Translational Lung Research, Division of Pulmonary Sciences and Critical Care Medicine, University of Colorado School of MedicineAurora, Colorado; 2 Division of Renal Diseases and Hypertension, University of Colorado School of MedicineAurora, Colorado; 3 Denver Health Medical CenterDenver, Colorado

**Keywords:** Acute kidney injury, heparan sulfate, heparanase, heparin, mice, sepsis

## Abstract

Heparanase, a heparan sulfate-specific glucuronidase, mediates the onset of pulmonary neutrophil adhesion and inflammatory lung injury during early sepsis. We hypothesized that glomerular heparanase is similarly activated during sepsis and contributes to septic acute kidney injury (AKI). We induced polymicrobial sepsis in mice using cecal ligation and puncture (CLP) in the presence or absence of competitive heparanase inhibitors (heparin or nonanticoagulant *N*-desulfated re-*N*-acetylated heparin [NAH]). Four hours after surgery, we collected serum and urine for measurement of renal function and systemic inflammation, invasively determined systemic hemodynamics, harvested kidneys for histology/protein/mRNA, and/or measured glomerular filtration by inulin clearance. CLP-treated mice demonstrated early activation of glomerular heparanase with coincident loss of glomerular filtration, as indicated by a >twofold increase in blood urea nitrogen (BUN) and a >50% decrease in inulin clearance (*P* < 0.05) in comparison to sham mice. Administration of heparanase inhibitors 2 h prior to CLP attenuated sepsis-induced loss of glomerular filtration rate, demonstrating that heparanase activation contributes to early septic renal dysfunction. Glomerular heparanase activation was not associated with renal neutrophil influx or altered vascular permeability, in marked contrast to previously described effects of pulmonary heparanase on neutrophilic lung injury during sepsis. CLP induction of renal inflammatory gene (IL-6, TNF-α, IL-1β) expression was attenuated by NAH pretreatment. While serum inflammatory indices (KC, IL-6, TNF-α, IL-1β) were not impacted by NAH pretreatment, heparanase inhibition attenuated the CLP-induced increase in serum IL-10. These findings demonstrate that glomerular heparanase is active during sepsis and contributes to septic renal dysfunction via mechanisms disparate from heparanase-mediated lung injury.

## Introduction

Since the original descriptions of “putrefaction” by Hippocrates, sepsis has been consistently recognized as a major source of human suffering and mortality (Majno 1991; Baron et al. 2006). Today, sepsis and its associated infections remain the most common cause of death in intensive care units worldwide (Vincent et al. 2009). Despite advances in our understanding of sepsis pathogenesis, clinical trials of sepsis therapeutics have been repeatedly disappointing, fueling a general sense of pessimism throughout the sepsis research community. Editorialists, however, have urged a “redoubling of efforts to seek new approaches to treatment that are based on a more crystalline view of the biology of sepsis” (Wenzel and Edmond 2012).

Using mechanistic animal and human studies, we recently demonstrated the importance of the pulmonary endothelial glycocalyx to lung function during sepsis (Schmidt et al. 2012). The glycocalyx is a heparan sulfate (HS)-rich layer of glycosaminoglycans and associated proteoglycans lining the vascular lumen. Sepsis is associated with activation of pulmonary heparanase, a HS-specific glucuronidase, leading to degradation of the pulmonary endothelial glycocalyx with consequent endothelial dysfunction and inflammatory lung injury (characteristic of the acute respiratory distress syndrome [ARDS]) (Schmidt et al. 2012). The relevance of heparanase activation to nonpulmonary organ dysfunction during sepsis, however, remains unexplored.

Similar to ARDS, sepsis-induced acute kidney injury (AKI) is common and of great clinical significance, yet remains poorly understood (Uchino et al. 2005; Zarjou and Agarwal 2011). AKI and ARDS frequently coexist in critically ill patients, suggesting a shared pathophysiology (Chien et al. 2004; Liu et al. 2007; Vincent 2011). We therefore hypothesized that sepsis is associated with activation of glomerular heparanase, contributing to the early onset of septic AKI. Using a robust animal model of polymicrobial sepsis and multiple measures of glomerular filtration, we demonstrate that glomerular heparanase activation is present in early sepsis and contributes to septic kidney dysfunction, via mechanisms disparate from those previously implicated in heparanase-mediated septic ARDS.

## Methods

### Materials/animals

We purchased *N*-desulfated re-*N*-acetylated heparin (NAH) from Iduron (Manchester, UK) and heparin from Moore Medical (Farmington, CT). We purchased Evans blue dye (EBD), bovine serum albumin (BSA), fluorescein isothiocyanate (FITC)-inulin (Cat # F3272-1G), heparinase-III (H8891), and HS (H7640) from Sigma (St. Louis, MO). We purchased Intramedic PE-10 tubing from Beckton-Dickinson (Franklin Lakes, NJ). We purchased male C57BL/6 mice (8–10 weeks old) from The Jackson Laboratory (Bar Harbor, ME). Animals were maintained in standard housing at the University of Colorado Anschutz Medical Center with ad lib access to food and water. Animals of identical age and housing (i.e., littermates) were randomly assigned to experimental or control groups. The Institutional Animal Care and Use Committee of the University of Colorado approved all mouse protocols. The care and handling of animals were in accord with National Institutes of Health guidelines for ethical animal treatment.

### Septic AKI model

We performed mouse cecal ligation and puncture (CLP) as previously described (Schmidt et al. 2012). Briefly, we anesthetized mice with isoflurane and externalized the cecum through a 1 cm abdominal incision. In CLP mice, we ligated 50% of the cecum with 4:0 silk suture, then punctured it through-and-through with a 23-gauge needle. After manually expressing stool from the puncture sites, we reinternalized the cecum. In sham mice, we externalized and then immediately reinternalized the cecum (without ligation or puncture). We closed the incision with 4:0 silk sutures and glue. We immediately resuscitated mice with 1-mL warmed subcutaneous saline and administered subcutaneous buprenorphine (0.05 μg/g body weight) for pain. Select mice were pretreated with saline, 150 μg NAH, or 5 units heparin (administered via 200 μL subcutaneous injection) 2 h prior to CLP. All assessments of AKI and/or the systemic inflammatory response were performed beginning 4 h after CLP/sham surgery.

### Hemodynamic assessment

We measured left ventricular pressures and heart rate in open-chest mice. After completion of the experimental protocol, anesthesia was achieved via intraperitoneal ketamine (100 μg/g body weight) and xylazine (15 μg/g body weight). We ventilated mice (MiniVent, Harvard Apparatus, Holliston MA) with 10 μL/g body weight tidal volumes at 120 breaths/min via tracheostomy. We performed a sternotomy and measured left ventricular pressure and heart rate by direct ventricular puncture with a Millar catheter (ADInstruments, Colorado Springs, CO).

### Serum assays

After completion of the experimental protocol, we collected mouse serum via direct cardiac puncture during organ harvest. Blood urea nitrogen (BUN) and serum creatinine were measured by quantitative colorimetric assays (DIUR-500 and DICT-500; BioAssay Systems, Hayword, CA). Serum IL-6, KC, TNF-α, IL-10, and IL-1β were measured via a multiplex immunoassay (K15012C-2; Meso Scale Discovery, Rockville, MD). We measured serum angiotensin II by enzyme-linked immunosorbent assay (ELISA) after phenyl extraction (400048, Cayman Chemical, Ann Arbor, MI), as per the manufacturer's instructions (589301, Cayman).

### Urine assays

After completion of the experimental protocol, urine was collected beginning 2 h prior to organ harvest. Urine protein (#500; Bio-Rad, Hercules, CA) and creatinine (BioAssay Systems, as above) were measured via colorimetric assays. We measured urine HS degradation activity (GWB-F49F83; GenWay Biotech, San Diego, CA) as previously described (Schmidt et al. 2012). Urinary IL-6 was measured by ELISA (M6000B; R&D Systems, Minneapolis, MN). We measured urinary nitric oxide species (NOx) using a colorimetric assay of urine nitrate (NO_3_^−^) and nitrite (NO_2_^−^), as per the manufacturer's instructions (780001; Cayman). We measured urine angiotensin II by ELISA, as per the manufacturer's instructions (589301; Cayman).

### Glomerular filtration rate measurement

Glomerular filtration rate (GFR) was measured by inulin clearance, as previously described (Lorenz and Gruenstein 1999). Four hours after CLP or sham surgery, we anesthetized mice with intraperitoneal pentobarbital (60 μg/g body weight) and placed a jugular central venous catheter (PE-10). We infused 0.75% FITC-inulin in 2.25% BSA (in saline) at a rate of 0.5 μL/g body weight/min. After 1 h of infusion, two 30-min collections of urine were obtained via a bladder catheter and weighed for volume determination. Blood for plasma inulin determination was drawn between urine collections. FITC in plasma and urine samples was measured using a CytoFluor plate reader (BioTek Instruments, Winooski, VT). GFR was defined as [inulin_urine_] × (urine output per 30 min)/[inulin_plasma_].

### Histology

Organs were formalin-fixed, paraffin-embedded, and sectioned (4 μm) as previously described (Schmidt et al. 2012). An automated tissue stainer (Shandon Varistain Gemini ES, Thermo Scientific, Waltham, MA) performed hematoxylin and eosin (H&E) staining. We performed immunofluorescence for heparanase (1:1000, Ins-26-2; ProSpec, East Brunswick, NJ) and neutrophil Ly-6B.2 (1:300, clone MCA771G; AbD Serotec, Raleigh, NC) as previously described (Schmidt et al. 2012). As a positive control for Ly-6B.2 immunofluorescence, we harvested lungs from mice 2 h after treatment with 20 μg/g body weight intravenous lipopolysaccharide (LPS, *E*. *coli* 055:B5, L2880, Sigma) or 200 μL saline. Ten random images/slide were captured at 1 μm steps (40× objective, 1.4 numerical aperture), and Z-stack reconstructions was performed using Nikon Elements (Nikon, Melville, NY) (Yoshida et al. 2010). After images were randomized and blinded, we performed image analysis and quantification using Metamorph (Molecular Devices, Sunnyvale, CA), using isotype controls to threshold heparanase positivity. Intensity was defined as the number of pixels positive/image multiplied by average pixel intensity. We performed immunohistochemistry as previously described (Yoshida et al. 2010), using a primary antibody (3G10, 1:100; US Biological, Marblehead, MA) against neoepitopes exposed during HS degradation by heparinase-III (heparitinase), a bacterial analog of mammalian heparanase (Kato et al. 1998; Dull et al. 2012; Schmidt et al. 2012). We performed fluorometric terminal deoxynucleotidyl transferase dUTP nick end labeling (TUNEL) staining (DeadEnd, G3250; Promega, Madison, WI) according to the manufacturer's instructions.

### Assessment of renal vascular permeability

We dissolved 0.5% EBD in 4% BSA (in saline). Four hours after CLP, mice were anesthetized with intraperitoneal pentobarbital (60 μg/g body weight) and 20 μg/g body weight EBD-albumin was injected into the right external jugular vein, as previously described (Schmidt et al. 2008). One hour later, we performed a midline laparotomy, exposing the abdominal aorta and kidneys. We killed the anesthetized mice via rapid exsanguination and harvested the left kidney for wet/dry ratio measurement (Schmidt et al. 2008). After flushing the right renal vasculature via arterial injection of saline, we snap-froze the right kidney in liquid nitrogen. We later homogenized the right kidney in 1 mL phosphate buffered saline and digested for 18 h in 2 mL formamide at 60°C. We centrifuged the digests at 5000*g* for 30 min and measured EBD content (in comparison to a standard curve) using spectrophotometry at 620 nm wavelength (Schmidt et al. 2008).

### Protein and mRNA analysis

Kidneys were homogenized for protein or RNA extraction (RNeasy, Qiagen, Valencia, CA) as previously described (Yoshida et al. 2010). We determined kidney homogenate angiotensin II by ELISA (589301; Cayman) and normalized to total protein concentrations (#500; Bio-Rad). We performed western blotting using primary antibodies against heparanase (Ins-26-2, 1:1000; ProSpec) and GAPDH (2118, 1:5000; Cell Signaling, Danvers, MA). We carried out reverse transcription (Superscript III First-Strand Synthesis System, Invitrogen, Carlsbad, CA) and performed quantitative polymerase chain reaction (qPCR) as previously described (Schmidt et al. 2012), using primers for mouse TNF-α (Mm00443260_g1), IL-1β (Mm00434228_m1), and IL-6 (Mm00446190_m1) purchased from Invitrogen. Expression was normalized to both sham mice and the housekeeping gene cyclophilin A (Applied Biosystems, Carlsbad, CA) and was reported as 2^−ΔΔCt^ (Schmidt et al. 2012).

### Assessment of inflammatory effect of HS fragments

HS (5 μg/μL) was treated for 1 h in vitro with either heat-inactivated (100°C × 5 min) (Schmidt et al. 2012) or enzymatically active heparinase-III (10 mU/mL in 100 mmol/L sodium acetate and 50 mmol/L calcium acetate). This dose of heparinase-III approximates what has been previously demonstrated to degrade endothelial HS in vitro (Florian et al. 2003). After 1 h of degradation, the HS/heparinase-III mixture was heat-inactivated to stop enzymatic activity of heparinase-III, and the mixture was added (at 5 μg/μL) to mouse lung endothelial cell monolayers (isolated and grown to confluence as previously described [Schmidt et al. 2012]) for 5 h. Additional monolayers were treated with unheated HS to control for nonspecific effects of HS heating. After completion of the experimental protocol, endothelial cells were lysed, and TNF-α, IL-1β, and IL-6 expression was determined using PCR, as described above.

### Statistical analysis

Data are represented as means ± SEM (or means alone on scatter plots). We used Student's two-tailed *t*-test when comparing two groups. Multiple comparisons were performed by analysis of variance (ANOVA) with Bonferroni's post hoc testing. Non-normal data were logistically transformed prior to analysis. Differences were statistically significant if *P* < 0.05. We performed all calculations using Prism (GraphPad, La Jolla, CA).

## Results

### Heparanase is expressed and active within glomeruli and glomerular arterioles of septic mice

**Figure 1:**
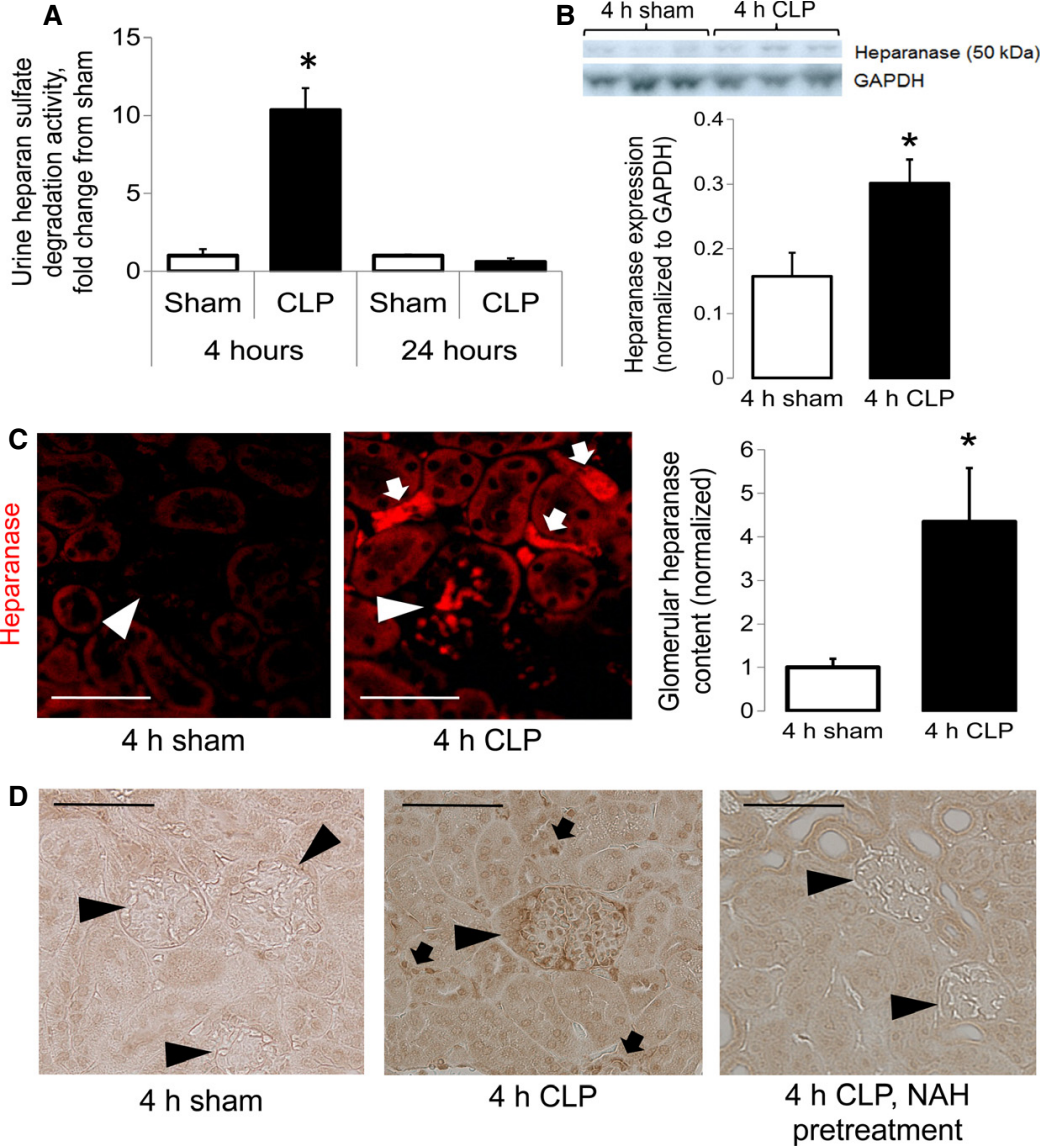
Cecal ligation and puncture (CLP) induces glomerular heparanase expression and activity. (A) Urine heparan sulfate (HS) degradation activity, a marker of heparanase activity, increases 4 h after CLP. This increase in activity normalizes 24 h after CLP (*n* = 4–5 per group, 4 h; *n* = 7–9 per group, 24 h). (B) The 4 h post-CLP increase in urine HS degradation activity coincides with increased renal heparanase expression, as demonstrated by western blot of kidney homogenates (*n* = 3 mice per group). (C) Heparanase expression after CLP is primarily localized to glomeruli (arrowheads) and periglomerular arterioles (arrows) (*n* = 7 mice per group). (D) Enzymatic activity of (peri)glomerular heparanase is further demonstrated by immunohistochemical evidence of HS cleavage in glomeruli (arrowheads) and periglomerular arterioles (arrows). Glomerular HS degradation is prevented by administration of the nonanticoagulant heparanase inhibitor NAH (150 μg in 200 μL subcutaneous saline) 2 h prior to CLP. Images in (C) representative of four mice per group. Scale bar for (C) and (D): 50 μm. *P < 0.05 for (A–C).

Urine HS degradation activity, a marker of renal heparanase activation (Rops et al. 2012; Schmidt et al. 2012), increased 4 h after CLP (Fig. 1A). This activity normalized within 24 h, suggesting that renal heparanase activation was an early event in the onset of experimental sepsis. Coincident with peak urinary HS degradation activity (i.e., 4 h after CLP), heparanase expression increased within glomeruli and surrounding arterioles (Fig. 1B and C). We additionally performed kidney immunohistochemistry using an antibody (3G10) targeted to remnant neoepitopes exposed after HS degradation (Dull et al. 2012). HS 3G10 immunostaining was noted within glomeruli and associated arterioles of mice 4 h after CLP (Fig. 1D). 3G10 immunostaining was prevented by pretreatment with the nonanticoagulant heparanase inhibitor NAH, demonstrating that septic glomerular HS degradation was heparanase dependent (Fig. 1D). Furthermore, these findings indicate that NAH pretreatment (at doses previously demonstrated to block pulmonary heparanase; Schmidt et al. 2012) was sufficient to inhibit glomerular heparanase activity. Of note, inhibitor experiments were not possible for urine HS degradation activity, due to heparin/NAH cross-reactivity with the assay.

### CLP induces early, heparanase-mediated renal dysfunction

**Figure 2:**
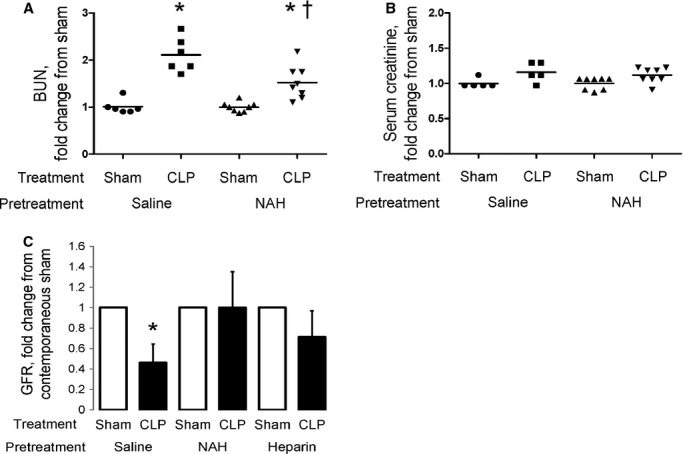
Heparanase inhibition attenuates cecal ligation and puncture (CLP)-induced loss of glomerular filtration rate (GFR). Four hours after CLP, (A) blood urea nitrogen (BUN) was significantly increased, suggesting loss of glomerular filtration (*n* = 4–5 per group). Serum creatinine was unchanged (B), consistent with the known insensitivity of this assay to septic acute kidney injury (AKI) (Doi et al. 2009) (*n* = 4–5 per group). (C) Four hour CLP-induced loss of glomerular filtration was confirmed by inulin clearance, a direct measure of GFR (*n* = 3–4 per group). Pretreatment with heparanase inhibitors *N*-desulfated re-*N*-acetylated heparin (NAH) (150 μg in 200 μL subcutaneous saline [A–C]) or heparin (5 units in 200 μL subcutaneous saline [C]) 2 h prior to CLP attenuated loss of glomerular filtration. *P < 0.05 compared to saline/sham; ^†^*P* < 0.05 compared to saline/CLP.

Kidney dysfunction occurred early in murine sepsis, coincident with the observed (Fig. 1) increase in glomerular heparanase expression and activity. Four hours after CLP, BUN was significantly elevated (Fig. 2A), indicative of decreased glomerular filtration. Serum creatinine was not elevated (Fig. 2B), reflecting the known insensitivity of creatinine as a marker of glomerular filtration during sepsis (Doi et al. 2009). CLP-induced loss of glomerular filtration was confirmed using FITC-inulin clearance, a highly sensitive measure of GFR (Fig. 2C).

Pretreatment of mice with NAH (at a dose sufficient to inhibit glomerular heparanase activity, Fig. 1D) attenuated CLP-induced changes in BUN and GFR (Fig. 2A and C). Furthermore, pretreatment with the anticoagulant heparanase inhibitor heparin (at doses shown to prevent heparanase activity in vivo; Schmidt et al. 2012) prevented CLP-induced loss of GFR (Fig. 2C). These findings demonstrate that glomerular heparanase activation contributes to renal dysfunction during early experimental sepsis.

### Glomerular heparanase activation occurs independently of renal neutrophilia or vascular leak

**Figure 3:**
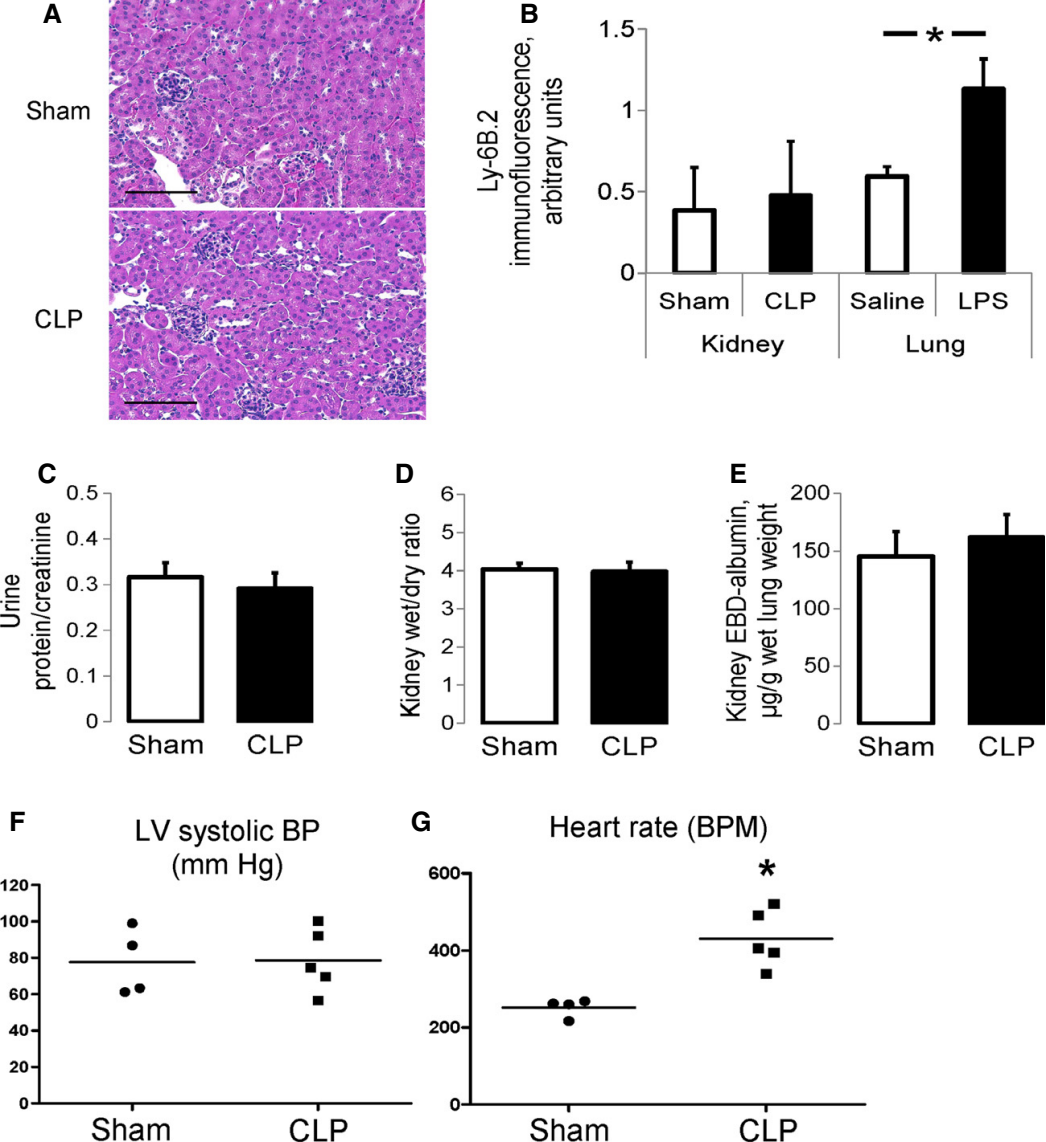
Early septic renal dysfunction occurs in the absence of renal neutrophilic inflammation, renal vascular leak, or systemic hypotension. (A) Hematoxylin and eosin staining of kidneys harvested 4 h after cecal ligation and puncture (CLP) or sham surgery revealed no evidence of acute tubular necrosis or neutrophilic inflammation. Images representative of five mice per group. Scale bar: 100 μm. (B) The absence of neutrophilic renal inflammation 4 h after CLP was confirmed via immunofluorescence for the neutrophil marker Ly-6B.2 (*n* = 7 mice per group). Lungs harvested from mice 2 h after intravenous lipopolysaccharide (LPS) (20 μg/g body weight) serve as a positive control for neutrophil infiltration (*n* = 4 mice per group). There was no evidence of renal vascular hyperpermeability 4 h after CLP, as demonstrated by unchanged (C) urine protein/creatinine ratios (*n* = 5 per group), (D) kidney wet–dry ratios (*n* = 7–8 per group), and (E) kidney Evans blue dye (EBD)-labeled albumin extravasation (*n* = 4–5 per group). (F) Left ventricular systolic pressures and (G) heart rates (measured via direct left ventricular puncture in anesthetized, ventilated, open-chest mice) were consistent with hemodynamically compensated, hyperdynamic sepsis 4 h after CLP. *P < 0.05 in (B–G).

We have previously demonstrated that septic activation of pulmonary endothelial heparanase induces neutrophil adhesion and extravasation, with consequent inflammatory septic lung injury (Schmidt et al. 2012). To our surprise, induction of glomerular heparanase activity 4 h after CLP occurred without any evidence of neutrophil influx, as demonstrated by unchanged H&E staining (Fig. 3A) and anti-Ly-6B.2 immunofluorescence (Fig. 3B). These findings suggest that heparanase mediates septic renal dysfunction through pathophysiologic processes distinct from septic lung injury.

As others have suggested that heparanase contributes to altered glomerular permeability (Szymczak et al. 2010; Salmon and Satchell 2012), we next sought to determine if septic induction of glomerular heparanase was coincident with renal endothelial barrier dysfunction. However, we found no evidence of altered renal endothelial permeability in early sepsis, as demonstrated by unchanged urine protein/creatinine ratios (Fig. 3C), kidney wet/dry ratios (Fig. 3D), and renal EBD-albumin extravasation (Fig. 3E) in control and septic mice.

Furthermore, there was little renal TUNEL staining 4 h after CLP (0.8 ± 0.1 cells/low powered field in sham [*n* = 4 mice]; 1.8 ± 0.5 cells/low powered field in CLP [*n* = 8 mice], 10 sections/mouse, *P* = 0.2). No histologic evidence of acute tubular necrosis (ATN) was apparent 4 h after CLP (Fig. 3A). Finally, CLP-treated mice demonstrated stable blood pressures but elevated heart rates (Fig. 3F and G), consistent with the hyperdynamic phase of sepsis typically associated with AKI onset (Zarjou and Agarwal 2011). These findings suggest that early septic glomerular heparanase activation (with concurrent loss of glomerular filtration) occurs independently of renal apoptosis or systemic hypoperfusion.

### NAH does not prevent the systemic inflammatory response to infection but attenuates serum IL-10

**Figure 4:**
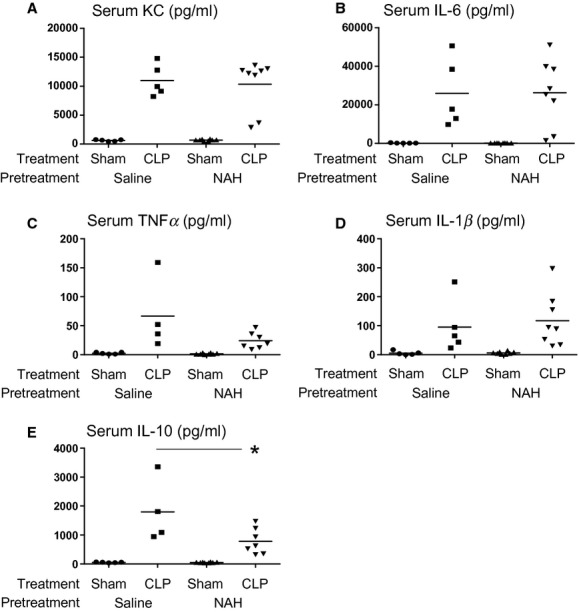
Impact of *N*-desulfated re-*N*-acetylated heparin (NAH) on the inflammatory response to infection. Pretreatment with the nonanticoagulant heparanase inhibitor NAH (150 μg in 200 μL subcutaneous saline 2 h prior to cecal ligation and puncture [CLP]) does not attenuate the systemic inflammatory response to CLP-induced peritonitis, as demonstrated by similar values of serum (A) KC, (B) IL-6, (C) TNF-α, and (D) IL-1β 4 h after CLP. In contrast, NAH pretreatment attenuated induction of the anti-inflammatory cytokine IL-10 4 h after CLP (E). *P < 0.05 compared to saline/CLP.

Given known effects of heparins in preventing neutrophil adhesion (Wang et al. 2005; Schmidt et al. 2012), we sought to determine if NAH pretreatment attenuated the inflammatory response to CLP. Pretreatment with NAH had no effect upon serum inflammatory indices KC, IL-6, TNF-α, and IL-1β 4 h after CLP (Fig. 4A–D), suggesting the renal-protective effects of NAH does not reflect a nonspecific attenuation of the systemic response to polymicrobial peritonitis. Interestingly, serum levels of the anti-inflammatory cytokine IL-10 were suppressed in the presence of heparanase inhibition, potentially reflecting a previously observed association between systemic heparanase administration and splenocyte IL-10 production (Bitan et al. 2010).

### Heparanase inhibition attenuates renal transcription of inflammatory mediators

**Figure 5:**
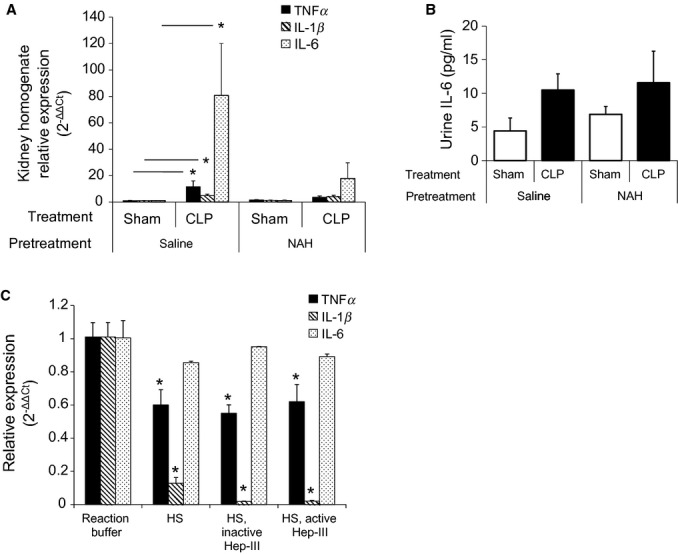
*N*-desulfated re-*N*-acetylated heparin (NAH) suppresses septic induction of renal inflammatory gene expression. (A) Four hours after cecal ligation and puncture (CLP), renal transcription of the inflammatory cytokines TNF-α, IL-1β, and IL-6 significantly increased. This induction did not occur in mice pretreated with the nonanticoagulant heparanase inhibitor NAH (150 μg in 200 μL subcutaneous saline) 2 h prior to CLP (*P < 0.05; *n* = 4–6 mice per group). (B) In contrast, NAH pretreatment did not attenuate urine IL-6 after CLP (*n* = 4–8 mice per group). (C) Exposure (5 h) of mouse lung microvascular endothelial cell monolayers to either full-length HS, HS treated for 1 h with inactivated heparinase-III, or HS digested for 1 h with the active heparanase analog heparinase-III (Hep-III) significantly decreased transcription of inflammatory cytokines TNF-α and IL-1β (*P < 0.05 compared to reaction buffer control; *n* = 4 per group). No alteration of IL-6 expression occurred (*n* = 2 per group).

CLP significantly increased renal transcription of the proinflammatory cytokines TNF-α, IL-1β, and IL-6 (Fig. 5A). In mice pretreated (2 h) with NAH, no statistically significant increase in the expression of these mediators occurred 4 h after CLP, suggesting that heparanase inhibition attenuates renal inflammatory gene expression (in the absence of neutrophilic infiltration, Fig. 3A). Interestingly, urinary IL-6 was not altered by NAH (Fig. 5B), indicating that (at least early in sepsis) urine IL-6 may reflect circulating levels of this cytokine as opposed to renal production.

We next sought to determine the mechanism by which heparanase inhibition suppressed renal inflammatory gene expression. Once fragmented, glycosaminoglycans (such as hyaluronic acid) may function as a proinflammatory damage-associated molecular pattern (Jiang et al. 2005). We sought to determine if HS fragmentation similarly induced activation of cellular inflammatory responses, thereby explaining the renal-protective effects of heparanase inhibition. Interestingly, both full-length and heparinase-III-treated HS suppressed inflammatory gene expression in endothelial monolayers (Fig. 5C). These findings, which contrast the inflammatory phenotype of low-weight hyaluronic acid, are consistent with previous observations suggesting that heparanase-degraded HS suppresses inflammatory responses (Lider et al. 1995).

### NAH does not influence renal NO or angiotensin II during early sepsis

**Figure 6:**
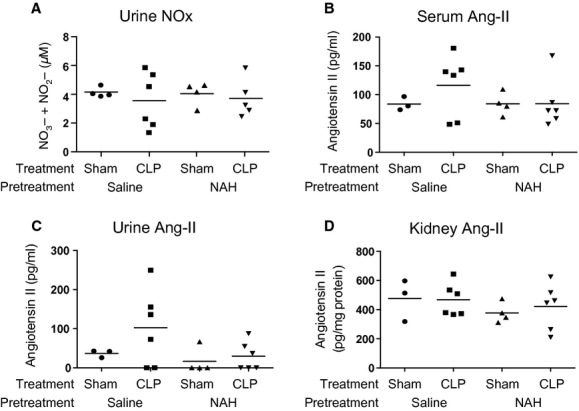
*N*-desulfated re-*N*-acetylated heparin (NAH) did not alter local or systemic vasoactive mediators after cecal ligation and puncture (CLP). (A) Urine nitrate (NO_3_^−^) and nitrite (NO_2_^−^) concentrations (NOx) were not significantly altered by CLP in the presence or absence of the nonanticoagulant heparanase inhibitor NAH (150 μg in 200 μL subcutaneous saline 2 h prior to CLP or sham surgery). Similarly, we were unable to detect statistically significant differences in serum (B), urine (C), or kidney (D) concentrations of the vasoconstrictor angiotensin II (Ang-II).

HS degradation may alter endothelial NO signaling (Florian et al. 2003), potentially altering glomerular arteriolar tone and thus glomerular filtration. We sought to measure renal NO production 4 h after CLP and to determine if this production was altered by antecedent NAH treatment. CLP was not associated with a significant change in urine nitrate and nitrite (NOx, Fig. 6A) species, similar to observations made in rats 24 h after CLP (Xie et al. 2008). NAH pretreatment did not influence urine NOx in CLP or sham-treated mice. Similarly, NAH did not alter serum, urine, or renal concentrations of the arteriolar vasoconstrictor angiotensin II (Fig. 6B–D) 4 h after CLP or sham surgery.

## Discussion

We have recently shown that pulmonary endothelial heparanase contributes to the onset of septic ARDS (Schmidt et al. 2012). We now expand these findings beyond lung injury, demonstrating that glomerular heparanase is induced in experimental sepsis and contributes to the onset of septic renal dysfunction. In contrast to septic ARDS, however, glomerular heparanase activation was not associated with neutrophilic renal inflammation, suggesting that the pathophysiologic mechanisms underlying heparanase-associated injury are organ specific.

To investigate renal heparanase activation during sepsis, we chose to employ CLP, the gold-standard model for experimental sepsis (Rittirsch et al. 2008). We found that urinary indices of heparanase activation were maximal 4 h after CLP (Fig. 1A), coincident with the so-called “hyperdynamic” phase of sepsis (normal arterial blood pressure, elevated heart rate, Fig. 3F and G) often characteristic of AKI onset (Bellomo et al. 2011). Indeed, this 4 h time-point coincided with renal dysfunction, as demonstrated by diminished GFR (increased BUN and decreased inulin clearance; Fig. 2) and local induction of inflammatory gene expression (Fig. 5A). The absence of gross ATN or neutrophilic inflammation in our model was surprising yet consistent with others’ observations of septic AKI (Langenberg et al. 2008; Lee et al. 2012). Indeed, even in severe (fatal) human sepsis, ATN was not widespread and did not account for the severity of kidney injury (Takasu et al. 2013). While others have described renal tubular apoptosis 24 h after CLP (Lee et al. 2012), we found no evidence of increased TUNEL staining 4 h after CLP, suggesting that tubular apoptosis may be a later event in septic AKI pathophysiology. Alternatively, apoptosis may not be necessary for septic AKI, as suggested by the paucity of renal cell death in fatal sepsis (Takasu et al. 2013).

Importantly, CLP was unable to induce renal dysfunction or inflammatory gene expression in mice pretreated with heparanase inhibitors (heparin, NAH). While pharmacologic inhibitors may have unanticipated nonspecific effects, we are reassured by the lack of NAH interference with the systemic inflammatory response to infection (Fig. 4A–D) as well as the finding that NAH effectively inhibits glomerular HS degradation (Fig. 1D). Given these protective effects of competitive inhibitors of heparanase enzymatic activity, we anticipate that heparanase contributes to septic AKI via degradation of glomerular HS. Within the pulmonary vasculature, loss of HS is associated with neutrophilic inflammation (Schmidt et al. 2012). However, we (Fig. 3B) and others (Lee et al. 2012) have demonstrated that septic AKI is not a neutrophilic injury – therefore, the mechanisms underlying heparanase-mediated kidney injury are likely distinct from those underlying heparanase-mediated ARDS.

The heparanase-related mechanisms specific to septic renal dysfunction remain unclear. While heparanase activity augmented renal inflammatory gene expression (Fig. 5A), this effect was not directly related to the elaboration of HS fragments, which we (Fig. 5C) and others (Lider et al. 1995) have found to be anti-inflammatory. While glomerular HS may participate in the glomerular barrier to fluid and protein (e.g., heparanase may contribute to the proteinuria characteristic of chronic kidney diseases) (Szymczak et al. 2010; Salmon and Satchell 2012), the relevance of this barrier function to septic AKI is uncertain, as we found no evidence of endothelial hyperpermeability (no change in kidney wet/dry ratio, urine protein/creatinine ratio, or kidney EBD-albumin extravasation) 4 h after CLP (Fig. 3). Finally, given the ability of endothelial surface HS to regulate NO production (Florian et al. 2003), the septic induction of heparanase activity could potentially alter afferent and/or efferent glomerular arteriole tone, leading to loss of GFR (Zarjou and Agarwal 2011). This potential mechanism is particularly intriguing, given recent literature emphasizing the role of glomerular arteriolar vasodilation in septic AKI onset (Bellomo et al. 2011). While heparanase inhibition did not alter urinary NO species or concentrations of the vasoconstrictor angiotensin II (Fig. 6), such indices of vascular tone may be insensitive to dynamic changes occurring within the afferent and/or efferent glomerular arterioles. Future translational investigations should pursue these and other potential mechanisms underlying heparanase-associated renal dysfunction, allowing for a better teleological understanding of the role of glomerular heparanase in renal physiology.

Interestingly, we found that NAH pretreatment attenuated CLP induction of serum IL-10 (Fig. 4E), an anti-inflammatory cytokine associated with the potentially deleterious counter anti-inflammatory response of sepsis (Adib-Conquy and Cavaillon 2009). This attenuation may be renal protective, as experimental depletion of IL-10 prevented AKI 24 h after CLP (Lee et al. 2012). The ability of NAH to suppress circulating IL-10 may in part reflect heparanase inhibition, as systemic heparanase exposure in vivo has been demonstrated to prime mouse splenocytes, augmenting IL-10 release (Bitan et al. 2010). Future studies should further investigate the mechanisms underlying organ-specific heparanase activity and systemic IL-10 release, as well as their impact on septic kidney injury.

Our study is limited by our inability to directly measure glomerular glycocalyx thickness in vivo. While we identify glomerular HS degradation during sepsis (Fig. 1A and D, consistent with a recent report [Xu et al. 2013]), it is unclear if this degradation occurs within the glomerular glycocalyx (Salmon et al. 2012), basement membrane (Sarrazin et al. 2011), or both. Techniques we have developed for direct visualization of the pulmonary endothelial glycocalyx (Yang et al. 2013) cannot be applied to the kidney, given the depth of glomeruli as well as the unique optical characteristics of the kidney capsule. We were similarly unable to identify mouse glycocalyx structures using in vivo confocal or two-photon microscopy (data not shown). Future multiphoton investigations using Munich–Wistar rats (a strain characterized by superficial renal glomeruli) may allow further mechanistic insight into the glomerular glycocalyx during sepsis (Salmon et al. 2012).

As we (Fig. 1) and others (Rops et al. 2012) have demonstrated that glomerular heparanase activity may be readily detected in urine, our findings have potential clinical significance. Detection of urinary heparanase activity could serve as a prognostic biomarker in sepsis, identifying patients at risk for AKI onset and progression. Given the significant mortality associated with septic AKI, this detection could improve patient outcomes by identifying a subset of septic patients who would benefit from the early administration of heparin, as suggested in both animal (Yang and Hauptman 1994) and human (Zarychanski et al. 2008) studies. Future human studies of urinary heparanase activity in the critically ill could therefore have significant clinical impact in sepsis, addressing a major need in critical care medicine.

## Conclusions

Our data demonstrate that glomerular heparanase activity increases in early murine sepsis and mediates the onset of renal dysfunction. The mechanisms underlying heparanase-mediated renal dysfunction are disparate from those underlying heparanase-mediated ARDS, demonstrating pathophysiologic heterogeneity of organ injury during sepsis.
